# Using machine learning to identify gene interaction networks associated with breast cancer

**DOI:** 10.1186/s12885-022-10170-w

**Published:** 2022-10-17

**Authors:** Liyuan Liu, Wenli Zhai, Fei Wang, Lixiang Yu, Fei Zhou, Yujuan Xiang, Shuya Huang, Chao Zheng, Zhongshang Yuan, Yong He, Zhigang Yu, Jiadong Ji

**Affiliations:** 1 Department of Breast Surgery, The Second Hospital, Cheeloo College of Medicine, Shandong University, 250033 Jinan, China; 2 School of Mathematics, Shandong University, Jinan, 250100 China; 3 Institute for Financial Studies, Shandong University, Jinan, 250100 China; 4 Institute of Translational Medicine of Breast Disease Prevention and Treatment, Shandong University, Jinan, 250100 China; 5 Department of Biostatistics, School of Public Health, Cheeloo College of Medicine, Shandong University, Jinan, 250012 China

**Keywords:** Breast cancer, Gene interaction network, Single nucleotide polymorphism, Differential network analysis

## Abstract

**Background:**

Breast cancer (BC) is one of the most prevalent cancers worldwide but its etiology remains unclear. Obesity is recognized as a risk factor for BC, and many obesity-related genes may be involved in its occurrence and development. Research assessing the complex genetic mechanisms of BC should not only consider the effect of a single gene on the disease, but also focus on the interaction between genes. This study sought to construct a gene interaction network to identify potential pathogenic BC genes.

**Methods:**

The study included 953 BC patients and 963 control individuals. Chi-square analysis was used to assess the correlation between demographic characteristics and BC. The joint density-based non-parametric differential interaction network analysis and classification (JDINAC) was used to build a BC gene interaction network using single nucleotide polymorphisms (SNP). The odds ratio (OR) and 95% confidence interval (95% CI) of hub gene SNPs were evaluated using a logistic regression model. To assess reliability, the hub genes were quantified by edgeR program using BC RNA-seq data from The Cancer Genome Atlas (TCGA) and identical edges were verified by logistic regression using UK Biobank datasets. Go and KEGG enrichment analysis were used to explore the biological functions of interactive genes.

**Results:**

Body mass index (BMI) and menopause are important risk factors for BC. After adjusting for potential confounding factors, the BC gene interaction network was identified using JDINAC. LEP, LEPR, XRCC6, and RETN were identified as hub genes and both hub genes and edges were verified. LEPR genetic polymorphisms (rs1137101 and rs4655555) were also significantly associated with BC. Enrichment analysis showed that the identified genes were mainly involved in energy regulation and fat-related signaling pathways.

**Conclusion:**

We explored the interaction network of genes derived from SNP data in BC progression. Gene interaction networks provide new insight into the underlying mechanisms of BC.

**Supplementary Information:**

The online version contains supplementary material available at 10.1186/s12885-022-10170-w.

## Background

The World Health Organization (WHO)'s International Agency for Research on Cancer (IARC) showed that the most predominant change in global cancer data in 2020 was a rapid increase in breast cancer (BC) incidence. BC has replaced lung cancer as the most common cancer worldwide [1]. The mortality rate of female BC is particularly high in transitional versus developed countries [2]. Obesity is a recognized risk factor for many cancers [3, 4]. Higher estrogen levels resulting from the aromatization of adipose tissue, increased production of inflammatory cytokines such as tumor necrosis factor α, interleukin-6, and prostaglandin E2, insulin resistance, and over activation of insulin-like growth factor signaling, adipokine production, and oxidative stress in obese women are associated with the development of cancer [5]. Structural variants of genes associated with BC and obesity, including LEP, LEPR, PON1, FTO, and MC4R, are associated with a higher or lower risk of BC [5].

Genome-wide association studies (GWAS) have linked many single nucleotide polymorphisms (SNPs) with BC occurrence [6–9]. In our previous studies, a potential relationship between the sequence variations of individual gene and BC has been proposed. In the study of 11 SNPs of PTPN1, rs3787345, rs718050, rs3215684, and rs718049 were associated with a reduction in BC risk [10]. Several studies have identified the genomic region of PTPN1 as a quantitative trait locus (QTL) in obesity and diabetes mellitus [11–13]. XRCC5 and XRCC6 SNP genotyping revealed that XRCC5 rs16855458 was associated with BC, XRCC6 rs2267437 was associated with ER-/PR- BC risk, and there may be interactions with environmental factors [14]. However, current research has largely focused on the impact of a single SNP on disease, and potential SNP-SNP interactions remain less well studied. Most diseases, including cancers, follow a polygenic model, indicating that they may involve multiple genes or SNPs [9]. However, little is known about how they interact. Understanding this issue will help to characterize the biological mechanism of BC risk.

Differential network analysis provides information about how genes interact. Recent studies suggest that cancer occurrence and development are not only caused by gene mutations but also by abnormal gene regulation [15]. Thus, it is important to assess the impact of both a single gene and gene–gene interactions on cancer onset and progression. Network analysis can effectively capture gene–gene interactions and genetic data can be used to establish gene regulation networks that characterize the biological mechanisms of disease [16]. A recent study analyzed the genetic and clinical data from gastric cancer patients using weighted gene co-expression network analysis (WGCNA) to explore new prognostic markers and therapeutic targets of gastric cancer [17]. Jubair et al. proposed a novel network-based method by integrating a protein–protein interaction network with gene expression data to identify biomarkers for different BC subtypes and predict patients ‘ survivability [18]. Another study constructed the multi-omics markers associated with BC by high-dimensional embedding and residual neural network [19]. To date, network analysis has relied on DNA methylation and RNA-seq data [17–20]. Meanwhile, genetic effects of combinations of functionally related SNPs may affect genes in a synergistic manner, thereby increasing BC risk [21, 22]. Network analysis using SNP data can provide insights into the mechanisms of disease.

The joint density-based nonparametric difference interaction network analysis and classification (JDINAC) method [23] was used to identify the differential gene interaction network between individuals in the BC and healthy control groups. Unlike previous studies, gene interaction network results were based on SNP data, providing new insight into potential pathogenic BC genes.

## Methods

### Participants

The study population has been described previously [10]. In brief, a hospital-based case–control study was used that included patients diagnosed with BC by pathology between April 2012 and April 2013 in the second hospital of Shandong University and 21 collaborative hospitals. Non-BC patients were selected as controls using 1:1 matching on age group (±3 years), hospital, and treatment time period (within 2 months). The subjects were 25 to 70 years of age. Patients with clinical or pathological diagnoses of recurrence or metastasis or other malignant tumor complications were excluded. The selection of cases and controls was carried out in strict accordance with project research design standards.

### Data collection

The data used for this study were obtained from a key project of clinical discipline dataset belonging to the hospitals under the Ministry of Health (administered) of the People's Republic of China [24]. The present study collected data from a face-to-face interview and, clinical breast and imaging examinations. The interview included questions relating to demographics, physiology, reproductive factors, chronic disease, and family history. Height, weight, hip and waist circumference were also obtained, body mass index (BMI) and the waist-hip rate (WHR) were calculated. Clinical examination results were also collected, including visual examination, palpation, and related diagnostic tests, including breast ultrasound, mammography, and blood testing. Blood samples were collected using an EDTA vacuum collector.

RNA-seq expression and clinical data from BC patients, including 112 tumor tissue samples and matched normal tissue samples, were downloaded from The Cancer Genome Atlas (TCGA; https://cancergenome.nih.gov/). SNP data from 4,030 and 3,494 women with and without BC, respectively, were screened using UK Biobank BC data [25]. These data were used as validation datasets.

### Genotyping and laboratory methods

The blood samples consisting of fasting venous whole blood were injected into EDTA anticoagulant tubes. These were placed fully upside-down in a 4 °C refrigerator and vertically placed in a -80 °C refrigerator after sedimentation. DNA was extracted using the Wizard Genomic DNA Purification Kit (a1120, Promega) and genotyped using the Sequenom MassARRAY SNP system (CapitalBio Technology, Beijing, China).

### Statistical analysis

#### Differential network analysis using JDINAC method

A Chi-square test was used to analyze differences in demographic and BC-related factors between the case and control groups. BMI data from the cases and controls was represented as the mean ± standard deviation. First, 101 SNPs were matched to their respective genes and the mean value of SNP for each gene was calculated for each sample. The gene difference interaction network was obtained using the JDINAC method. The 95% confidence interval (95% CI) and odds ratio (OR) were also estimated for hub gene polymorphisms in the gene difference interaction network. Significance was defined as a *p*-value < 0.05. All data were statistically analyzed using R × 64 4.1.0.

The JDINAC method assumes that the network-level difference between BC patients and healthy controls is the result of the collective effect of differential pairwise gene–gene interactions that are characterized by the conditional joint density of two genes [23]. Formally, *Y*_*l*_ (*l* = 1,2,…,n) is the binary response vector and if the *l*th subject is BC, *Y*_*l*_ = 1, otherwise *Y*_*l*_ = 0. Pr is the probability of the subject with BC, i.e., Pr = P(*Y*_*l*_ = 1), and *S*_*i*_ is the *i*th gene risk score. The JDINAC method based on the logistic regression is then represented as:

1 $$\text{logit(Pr)}={\alpha }_{0}+\sum_{t=1}^{T}{\alpha }_{t}{Z}_{t}+\sum_{i=1}^{p}\sum_{j>i}^{p}{\beta }_{ij}1\mathrm{n}\frac{{f}_{ij}^{1}\left({S}_{i},{S}_{j}\right)}{{f}_{ij}^{0}\left({S}_{i},{S}_{j}\right)}, s.t. \sum_{i=1}^{p}\sum_{j>i}^{p}\left|{\beta }_{ij}\right|\le c,c>0,$$

Z_t_ (t = 1,…,T) denotes covariates such as BMI and age, *p* is the number of genes. $f_{ij}^k\left(k=0,1\right)$  denotes the group conditional joint density of S_*i*_ and S_*j*_ for group *k*, respectively, i.e.,

2 $$\left(\left({S}_{i},{S}_{j}\right)\left|Y=1\right.\right)\sim {f}_{ij}^{1}$$

and

3 $$\left(\left({S}_{i},{S}_{j}\right)\left|Y=0\right.\right)\sim {f}_{ij}^{0}$$

which represents the strength of interaction between *S*_*i*_ and *S*_*j*_ for group *k* [23]*.* β_*ij*_ indicates the dependency between specific conditional groups.

JDINAC adopted a multiple randomly split algorithm to improve the accuracy and robustness of the results. A Lasso penalty was added to the logistics regression to estimate the coefficient β_*ij*_ and a cross-validation method was used to determine the best penalty parameter. The importance score for each pair $S_i,S_j$ was obtained by the following formula:

4 $${\omega }_{ij}=\sum_{t=1}^{T}I\left({\widehat{\beta }}_{ij,t}\ne 0\right), i,j=1,\dots ,p, j>i$$

where $\omega_{ij}$  was the importance score, $I\left(\cdot\right)$  was an indicative function, ${\widehat\beta}_{ij,t}\left(t=1,\dots,T\right)$ was the *t*th estimation of the coefficient $\beta_{ij}$ . The importance scores represented the differential dependency weight of each pair $\left(S_i,S_j\right)$  between two groups [23]. The difference network was inferred by connecting pairs with high importance scores through their shared genes.

#### Differential expression analysis and enrichment analysis

The edgeR package [26] was utilized to identify differentially expressed genes in TCGA breast cancer data to test the reliability of the JDINAC results. Multiplicity correction was performed by applying the Benjamini–Hochberg method on the *p*-values.

To explore the biological functions of the identified interaction genes, Gene Ontology (GO) and Kyoto Encyclopedia of Genes and Genomes (KEGG) pathways in enrichment analysis were performed by the R package "clusterProfiler" [27]. Only terms with a multiple-test adjusted *p*-value < 0.05 were considered significant.

## Results

### Participant demographic and lifestyle characteristics

There were 1,916 subjects in the study, including 953 and 963 in the BC and control groups, respectively. There were significant differences in BMI and menopausal status between the two groups (*p*-value < 0.05) (Table 1). Women with BC had a higher BMI than that of healthy women (24.36 ± 3.46 vs. 24.01 ± 3.11, respectively), indicating that obesity may be a risk factor for BC.

**Table 1:** Clinical characteristics of the study population

Variables	Control n (%)	BC case n (%)	*X*^2^	*p* value
Age, y			3.563	0.468
25-	76(7.89)	62(6.51)		
35-	329(34.16)	302(31.69)		
45-	352(36.55)	364(38.2)		
55-	183(19)	200(20.99)		
65-	23(2.39)	25(2.62)		
BMI, kg/m^2^			6.412	0.011
≤ 28	849(90.90)	799(87.23)		
> 28	85(9.10)	117(12.77)		
WHR			3.344	0.067
< 0.85	458(53.82)	389(49.30)		
≥ 0.85	393(46.18)	400(50.70)		
Age at menarche, y			1.036	0.596
7–11	16(1.66)	11(1.15)		
12–13	231(24.01)	223(23.4)		
≥ 14	715(74.32)	719(75.45)		
Number of births			0.501	0.479
0	25(2.63)	20(2.13)		
≥ 1	926(97.37)	918(97.87)		
Diabetes mellitus history			0.094	0.759
Yes	32(3.36)	34(3.62)		
No	921(96.64)	906(96.38)		
Plasma glucose, mM			0.593	0.441
< 7	739(76.22)	776(95.45)		
≥ 7	29(3.78)	37(4.55)		
Smoking			2.406	0.121
Yes	10(1.04)	18(1.89)		
No	950(98.96)	932(98.11)		
Alcohol consumption			3.089	0.079
Yes	3(0.31)	9(0.95)		
No	956(99.69)	939(99.05)		
Menopause			6.251	0.012
Yes	260(28.11)	309(33.48)		
No	665(71.89)	614(66.52)		
Cholesterol, mmol/L			0.239	0.625
≤ 5.18	505(70.53)	500(69.35)		
> 5.18	211(29.47)	221(30.65)		

### Differential network of gene interaction

**Fig. 1:**
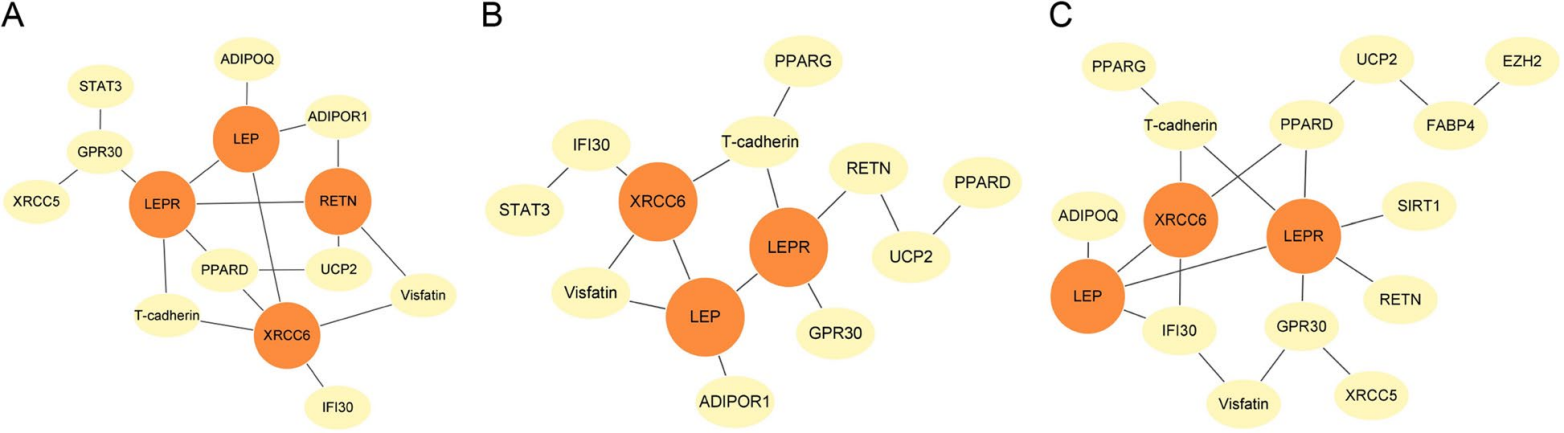
The differential interaction networks inferred by the joint density-based nonparametric difference interaction network analysis and classification (JDINAC). The hub genes are colored orange. **A** no adjustment for covariates. **B** adjustment for BMI. **C** adjustment for the menopause status

Twenty genes that might be related to the pathogenesis of BC and 101 SNPs associated with these genes were selected. The differential gene interaction network was estimated based on four scenarios: no adjustment for covariates, adjustment for BMI, adjustment for the menopause status (Fig. 1), and adjustment for BMI and menopause status simultaneously (see Additional file 1). The number of edges selected under the four scenarios was 18, 14, 19 and 16, respectively. The orange nodes in the figure represent the central genes with at least four adjacent genes in the network. All scenarios had the three genes, LEP, LEPR, and XRCC6 in common. Gene pairs were ranked based on the importance scores derived from JDINAC and the top ten pairs in the network with no covariate adjustment are summarized in Table 2. Among them, six pairs had evidence of interaction in STRING database [28]. Additional data are shown in Additional files 2, 3, 4 and 5.

**Table 2:** Top 10 gene interaction pairs identified by JDINAC with no covariate

	Gene1	Gene2	Importance scores	STRING	
1	PPARD	UCP2	13	Y	
2	LEP	XRCC6	12	N	
3	LEP	LEPR	11	Y	
4	LEPR	RETN	10	Y	
4	T-cadherin	XRCC6	10	N	
6	IFI30	XRCC6	9	N	
7	LEPR	T-cadherin	8	N	
7	VISFATIN	XRCC6	8	N	
9	GPR30	XRCC5	6	N	
10	ADIPOQ	LEP	5	Y	
10	ADIPOR1	RETN	5	Y	
10	GPR30	STAT3	5	N	
10	RETN	UCP2	5	Y	

Y indicates that the pair of genes has an interaction in the STRING database, and N indicates not

### Association between polymorphisms and BC risk

Next, the association between SNPs in the hub genes of differential networks and BC risk was assessed (Table 3). Most SNPs were not associated with BC significantly. Rs1137101 (OR = 0.728, *p*-value = 0.002) and rs4655555 (OR = 0.825, *p*-value = 0.015) contained in LEPR were significantly associated with BC risk, while the LEP, XRCC6, and RETN polymorphisms were not significantly. Functional consequences of SNPs on genes were also shown in Table 3. Rs4655555 is an intron variant. Rs1137101 is a missense variant and coding sequence variant reported as benign [29].

**Table 3:** The association of SNPs in hub genes with breast cancer (BC) adjusted for BMI and menopause status

SNP IDs	Gene	CHR	Alleles	OR	95% CI	*p* value	Functional consequence
rs2167270	LEP	7	G > A	1.007	0.851–1.191	0.937	5_prime_UTR_variant
rs4731426	LEP	7	C > G	0.991	0.846–1.161	0.911	intron_variant
rs10487506	LEP	7	A > G	0.970	0.829–1.135	0.702	upstream_transcript_variant,2KB_upstream_variant
rs10954173	LEP	7	G > A	0.998	0.846–1.178	0.981	intron_variant
rs3828942	LEP	7	A > G	0.985	0.843–1.151	0.854	intron_variant
rs4655555	LEPR	1	A > T	0.825	0.706–0.934	0.015	intron_variant
rs10244329	LEPR	1	A > T	0.971	0.830–1.136	0.715	intron_variant
rs1137101	LEPR	1	G > A	0.728	0.598–0.885	0.002	missense_variant, coding_sequence_variant
rs1137100	LEPR	1	G > A	0.956	0.810–1.128	0.595	missense_variant, coding_sequence_variant
rs3745369	RETN	19	G > C	1.085	0.945–1.247	0.246	500B_downstream_variant
rs34861192	RETN	19	G > A	0.975	0.813–1.170	0.789	2KB_upstream_variant, upstream_transcript_variant
rs3219175	RETN	19	G > A	0.964	0.728–1.273	0.794	2KB_upstream_variant, upstream_transcript_variant
rs3219177	RETN	19	C > T	1.011	0.716–1.428	0.949	intron_variant
rs34124816	RETN	19	A > C	1.168	0.926–1.476	0.190	2KB_upstream_variant, upstream_transcript_variant
rs1862513	RETN	19	C > G	1.083	0.941–1.247	0.265	2KB_upstream_variant, upstream_transcript_variant
rs3745367	RETN	19	G > A	0.969	0.844–1.113	0.657	intron_variant
rs2267437	XRCC6	22	C > G	0.985	0.843–1.151	0.851	intron_variant, upstream_transcript_variant,2KB_upstream_variant
rs2284082	XRCC6	22	T > C	0.973	0.852–1.111	0.683	intron_variant
rs5751129	XRCC6	22	T > C	0.903	0.726–1.120	0.353	intron_variant, upstream_transcript_variant,2KB_upstream_variant
rs5751131	XRCC6	22	A > G	0.995	0.871–1.136	0.938	intron_variant

### Identification of the interaction network

RNA-seq expression and clinical data from BC patients were obtained from TCGA to analyze and verify the identified hub genes. The validation dataset included 112 subjects for whom both tumor and matched normal samples were available. All genes available in the TCGA dataset were analyzed to detect differences between tumor and normal samples, and 10 common genes in Fig. 1 were screened out from the results. LEP, LEPR and XRCC6 expression was significantly different between two groups (Table 4). RETN was not differentially expressed in the TCGA data.

**Table 4:** The validation results of the 10 identical genes in Fig. 1 using TCGA data

Gene	logFC	logCPM	*p* value	p-adjust
LEPR	-2.52777	5.193642	1.65 × 10^–39^	8.38 × 10^–38^
LEP	-5.98334	7.009349	2.35 × 10^–32^	5.20 × 10^–31^
T-cadherin	-1.17561	4.687897	7.96 × 10^–23^	6.45 × 10^–22^
IFI30	0.872733	-0.95925	8.69 × 10^–11^	2.42 × 10^–10^
UCP2	0.827575	6.632093	1.06 × 10^–9^	2.71 × 10^–9^
PPARD	0.328611	4.92447	1.74 × 10^–6^	3.41 × 10^–6^
XRCC6	0.276328	7.708723	3.52 × 10^–6^	6.70 × 10^–6^
GPR30	-0.79614	2.56532	0.000122	0.000203
RETN	0.10441	-3.79534	0.683576	0.714306
Visfatin	-0.01691	6.395228	0.866491	0.881913

logFC, log_2_ fold-change; logCPM, log_2_ counts-per-million

Genetic data from 4,030 BCs and 3,494 controls in the UK Biobank was used to verify the eight identical edges of the three networks in Fig. 1 using logistic regression. The data were randomly divided into two parts, the kernel density function of the BC and control groups were estimated, and logistic regression was used to assess the corresponding *p*-value of the eight edges (Table 5). The results showed that the first four edges were significantly different (*p*-value < 0.05). The genes connected by these four edges were the identified hub genes, indicating that the interaction between hub genes in this network is more significant than it is for other genes.

**Table 5:** The validation results of the 8 identical edges in Fig. 1 using UK Biobank data

Gene1	Gene2	*p* value
LEP	XRCC6	0.047
LEP	LEPR	0.005
LEPR	RETN	0.002
GPR30	LEPR	0.010
IFI30	XRCC6	0.206
T-cadherin	XRCC6	0.052
LEPR	T-cadherin	0.051
PPARD	UCP2	0.318

### Enrichment analysis

**Fig. 2:**
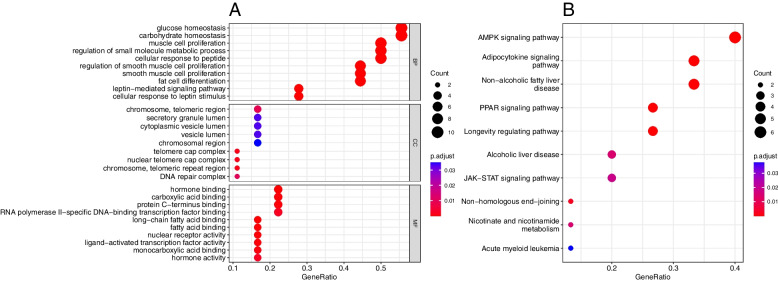
GO function and KEGG pathway enrichment analysis of the genes identified by JDINAC. **A** Dot plots show the top ten enriched GO BP, CC, and MF terms for identified genes; **B** Dot plots show the top ten enriched KEGG pathways. BP, Biological Processes; CC, Cell Component; MF, Molecular Function

GO analysis showed that the biological processes of the identified genes were mainly related to glucose homeostasis and carbohydrate homeostasis (Fig. 2). KEGG pathway analysis showed that these genes were mainly enriched in adenosine-monophosphate-activated protein kinase (AMPK) signaling pathway, adipocytokine signaling and non-alcoholic fatty liver disease (Fig. 2).

## Discussion

This study sought to identify potential pathogenic genes associated with BC by constructing a BC gene interaction network. This study extended the results of prior studies [14] by not only assessing the effect of a single gene on BC but also the gene interaction network, providing new insight into how genetic factors impact complex human diseases. These results suggest that BMI and menopausal status may be risk factors for BC. The gene interaction network obtained using the JDINAC method showed that LEPR, LEP, XRCC6, and RETN have significant interactivity difference between BC patients and healthy women, and are associated with higher BC risk. However, analysis of hub gene polymorphisms indicated that only LEPR rs1137101 and rs4655555 were strongly linked to BC. Other independent datasets and bioinformatics analysis tools were used to verify the hub genes and the edges, increasing the reliability of the results. The expression of LEPR, LEP and XRCC6 was significantly associated with BC in TCGA dataset. Meanwhile, UK Biobank SNP data validated their interaction on BC.

GO enrichment analysis showed that the interacting genes were closely related to cell energy and cell metabolism, such as glucose homeostasis, carbohydrate homeostasis, muscle cell proliferation and regulation of small molecules. The results in KEGG analysis were consistent with those by GO analysis. Studies have shown that AMPK is the main cellular energy sensor [30]. Reduced activity of AMPK is associated with altered cellular metabolic processes that drive BC tumor growth and progression. If AMPK is activated, it can respond to adenosine triphosphate (ATP) depletion, glucose starvation, and metabolic stress [31]. Obesity-related factors modulate metabolic pathways in BC, providing a molecular link between obesity and BC.

Many studies have shown that LEP and LEPR play an important role in obesity. LEP is a hormone secreted by adipose tissue, which regulates eating and energy consumption through the hypothalamic region of the brain [32]. Circulating leptin binds to LEPR, activating Janus kinase 2 (JAK2), phosphorylating three tyrosine residues in LEPR, and inducing phosphorylation of STAT transcription factors, STAT5 and STAT3, which are involved in the development of BC [32]. Leptin may stimulate the expression of estrogen by increasing aromatase expression, which is also involved in BC development [33]. The LEPR rs1137101 polymorphism results from a nonconservative A to G substitution at codon 223, reducing leptin binding and impairing signaling [34]. While the effect of LEPR rs4655555 on the development of BC has not yet been reported, one study has shown that rs4655555 is significantly correlated with plasma soluble leptin receptor levels and may inform diabetes prognosis [35]. The findings from the current study further support the evidence that LEP and LEPR play an important role in BC pathogenesis.

The impact of RETN on BC has been reported previously. RETN is highly expressed in BC tissues and may serve as a biomarker for disease stage and the degree of inflammation [36, 37]. Low-grade systemic inflammation is one of the characteristics of obesity [38], and RETN is shown to exert pro-inflammatory properties by upregulating pro-inflammatory cytokines [39] through the NFκB signaling pathway [40] that lead to inflammation and tumorigenesis. Several studies have also linked XRCC6 with an increased risk of BC [14, 41, 42]. Interaction between XRCC6 genetic polymorphisms and reproductive risk factors is thought by some researchers to contribute to estrogen exposure, which results in double-strand breaks on BRCA1 and BRCA2 DNA and induces BC [41]. XRCC6 is also involved in the production of proinflammatory cytokines induced by lipopolysaccharide (LPS) in human macrophages and monocytes. Proinflammatory cytokine production is, in turn, associated with obesity and BC [42].

Recent studies have used gene expression data to explore the pathogenesis of BC [18] and other diseases [17, 20]. However, no genetic interaction network has been constructed to identify potential BC pathology genes using SNP data. As discussed previously, single genetic variants often explain only a small fraction of phenotypic variation, that is, the problem of missing heritability [43]. Gene–gene interactions are proposed as a potential source of this problem [44]. The current study built gene interaction networks based on SNP data to explain the etiology of complex human traits. While high-throughput SNP genotyping methods have been developed, the computational and statistical challenges of simultaneously analyzing large SNP datasets still exist [9]. The method used here provides ideas for handling SNP data. In addition, because BC incidence is affected by demography [45, 46] the gene network was constructed adjust the influence of confounding factors such as BMI and menopause, making the results more reliable. This study does have some limitations, however. Only the interaction between paired genes was assessed. For BC, the relationship between genes may be more complicated. Future studies should assess more complex interactions associated with this disease.

## Conclusions

Potential pathogenic BC genes were investigated by constructing a gene interaction network. LEP, LEPR, XRCC6, and RETN had significant interactions during BC, and LEPR polymorphisms may also be associated with BC development. Gene network analysis can provide more detailed information about the pathogenesis of complex diseases.

## Supplementary Information

**Additional file 1:** **Figure S1.** The differential interaction network inferred by JDINAC after adjusting for BMI and menopause status.

**Additional file 2:** **Table S1.** Top 10 gene interaction pairs identified by JDINAC after adjusting for BMI.

**Additional file 3:** **Table S2.** Top 10 gene interaction pairs identified by JDINAC after adjusting for menopausal status.

**Additional file 4:** **Table S3.** Top 10 gene interaction pairs identified by JDINAC after adjusting for BMI and menopause status.

**Additional file 5:** **Table S4.** The association of IFI30 polymorphisms with BC adjusted for BMI and menopause status.

## Data Availability

The datasets analyzed during the current study are not publicly available due to privacy but are available from the corresponding author on reasonable request.

## Abbreviations

BC: Breast cancer
LEP: Leptin
LEPR: Leptin receptor
XRCC6: X-ray repair cross complementing 6
RETN: Resistin
JDINAC: The joint density-based non-parametric differential interaction network analysis and classification
OR: Odds ratio
CI: Confidence interval
SNP: Single nucleotide polymorphism
TCGA: The Cancer Genome Atlas
BMI: Body mass index
IARC: International Agency for Research on Cancer
GWAS: Genome wide association study
WGCNA: Weighted gene co-expression network analysis
WHR: Waist-hip rate
JAK2: Janus kinase 2
LPS: Lipopolysaccharide
