# Supplementary figures and images for: Using machine learning to identify gene interaction networks associated with breast cancer
Source: BMC Cancer. 2022 Oct 17;22:1070. doi: 10.1186/s12885-022-10170-w (PMC9575346; doi:10.1186/s12885-022-10170-w)

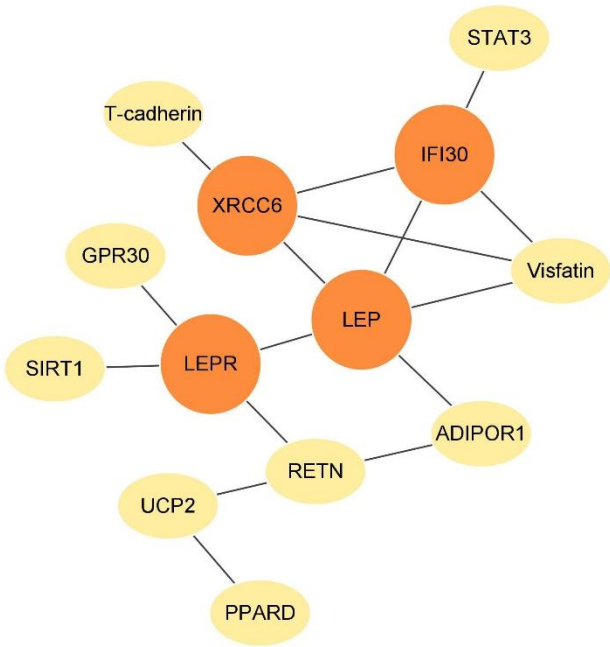

Supplement: Supplementary file 1 — Additional file 1: Figure S1. The differential interaction network inferred by JDINAC after adjusting for BMI and menopause status. [file 12885_2022_10170_MOESM1_ESM.pdf]
