# Supplementary material for: Using machine learning to identify gene interaction networks associated with breast cancer
Source: BMC Cancer. 2022 Oct 17;22:1070. doi: 10.1186/s12885-022-10170-w (PMC9575346; doi:10.1186/s12885-022-10170-w)
Supplement: Supplementary file 2 — Additional file 2: Table S1. Top 10 gene interaction pairs identified by JDINAC after adjusting for BMI. [file 12885_2022_10170_MOESM2_ESM.docx]

|  | Gene1 | Gene2 | Importance scores | STRING |
| --- | --- | --- | --- | --- |
| 1 | T-cadherin | XRCC6 | 20 | N |
| 2 | LEPR | RETN | 15 | Y |
| 3 | LEP | LEPR | 14 | Y |
| 4 | LEP | XRCC6 | 12 | N |
| 5 | ADIPOR1 | LEP | 8 | Y |
| 5 | GPR30 | LEPR | 8 | N |
| 5 | IFI30 | XRCC6 | 8 | N |
| 5 | VISFATIN | XRCC6 | 8 | N |
| 9 | PPARG | T-cadherin | 6 | N |
| 9 | LEP | VISFATIN | 6 | Y |
| 10 | IFI30 | STAT3 | 5 | N |
| 10 | LEPR | T-cadherin | 5 | N |

**Table S1.** Top 10 gene interaction pairs identified by JDINAC after adjusting for BMI

Y indicates that the pair of genes has an interaction in the STRING, and N indicates not.
