# Supplementary material for: Using machine learning to identify gene interaction networks associated with breast cancer
Source: BMC Cancer. 2022 Oct 17;22:1070. doi: 10.1186/s12885-022-10170-w (PMC9575346; doi:10.1186/s12885-022-10170-w)
Supplement: Supplementary file 3 — Additional file 3: Table S2. Top 10 gene interaction pairs identified by JDINAC after adjusting for menopausal status. [file 12885_2022_10170_MOESM3_ESM.docx]

|  | Gene1 | Gene2 | Importance scores | STRING |
| --- | --- | --- | --- | --- |
| 1 | LEP | XRCC6 | 20 | N |
| 2 | IFI30 | XRCC6 | 18 | N |
| 3 | LEPR | RETN | 12 | Y |
| 4 | LEP | LEPR | 10 | Y |
| 5 | PPARD | XRCC6 | 9 | N |
| 5 | T-cadherin | XRCC6 | 9 | N |
| 7 | IFI30 | LEP | 8 | N |
| 8 | PPARG | T-cadherin | 7 | N |
| 9 | LEPR | PPARD | 6 | N |
| 9 | LEPR | SIRT1 | 6 | N |
| 9 | PPARD | UCP2 | 6 | Y |
| 10 | EZH2 | FABP4 | 5 | N |
| 10 | ADIPOQ | LEP | 5 | Y |
| 10 | LEPR | T-cadherin | 5 | N |
| 10 | IFI30 | VISFATIN | 5 | N |
| 10 | GPR30 | XRCC5 | 5 | N |

**Table S2.** Top 10 gene interaction pairs identified by JDINAC after adjusting for menopausal status

Y indicates that the pair of genes has an interaction in the STRING, and N indicates not.
