# Supplementary material for: Using machine learning to identify gene interaction networks associated with breast cancer
Source: BMC Cancer. 2022 Oct 17;22:1070. doi: 10.1186/s12885-022-10170-w (PMC9575346; doi:10.1186/s12885-022-10170-w)
Supplement: Supplementary file 4 — Additional file 4: Table S3. Top 10 gene interaction pairs identified by JDINAC after adjusting for BMI and menopause status. [file 12885_2022_10170_MOESM4_ESM.docx]

|  | Gene1 | Gene2 | Importance scores | STRING |
| --- | --- | --- | --- | --- |
| 1 | LEP | XRCC6 | 16 | N |
| 2 | T-cadherin | XRCC6 | 14 | N |
| 3 | LEP | LEPR | 13 | Y |
| 3 | IFI30 | XRCC6 | 13 | N |
| 5 | LEPR | RETN | 11 | Y |
| 6 | VISFATIN | XRCC6 | 7 | N |
| 7 | RETN | UCP2 | 6 | Y |
| 8 | GPR30 | LEPR | 5 | N |
| 9 | ADIPOR1 | LEP | 4 | Y |
| 9 | IFI30 | LEP | 4 | N |
| 9 | ADIPOR1 | RETN | 4 | Y |
| 9 | LEPR | SIRT1 | 4 | N |
| 9 | IFI30 | STAT3 | 4 | N |
| 9 | PPARD | UCP2 | 4 | Y |
| 9 | IFI30 | VISFATIN | 4 | N |
| 9 | LEP | VISFATIN | 4 | Y |
| 10 | ADIPOR1 | GPR30 | 3 | N |
| 10 | GPR30 | PTP1B | 3 | N |
| 10 | LEPR | STAT3 | 3 | Y |
| 10 | RETN | STAT3 | 3 | Y |
| 10 | LEPR | T-cadherin | 3 | N |
| 10 | PPARD | T-cadherin | 3 | N |
| 10 | PPARG | T-cadherin | 3 | N |
| 10 | CETP | VISFATIN | 3 | N |
| 10 | LEPR | XRCC5 | 3 | N |
| 10 | FABP4 | XRCC6 | 3 | N |
| 10 | PPARD | XRCC6 | 3 | N |
| 10 | XRCC5 | XRCC6 | 3 | Y |

**Table S3.** Top 10 gene interaction pairs identified by JDINAC after adjusting for BMI and menopause status

Y indicates that the pair of genes has an interaction in the STRING, and N indicates not.
