# Supplementary material for: Using machine learning to identify gene interaction networks associated with breast cancer
Source: BMC Cancer. 2022 Oct 17;22:1070. doi: 10.1186/s12885-022-10170-w (PMC9575346; doi:10.1186/s12885-022-10170-w)
Supplement: Supplementary file 5 — Additional file 5: Table S4. The association of IFI30 polymorphisms with BC adjusted for BMI and menopause status. [file 12885_2022_10170_MOESM5_ESM.docx]

**Table S4.** The association of IFI30 polymorphisms with BC adjusted for BMI and menopause status

| SNP IDs | Gene | CHR | Alleles | OR | 95% CI | p value | Functional consequence |
| --- | --- | --- | --- | --- | --- | --- | --- |
| rs11554159 | IFI30 | 19 | G>A | 0.923 | 0.744-1.144 | 0.466 | missense_variant,  coding_sequence_variant |
| rs1045747 | IFI30 | 19 | T>C | 1.217 | 1.010-1.469 | 0.040 | 3_prime_UTR_variant |
| rs7125 | IFI30 | 19 | A>G | 1.051 | 0.902-1.226 | 0.523 | coding_sequence_variant,  synonymous_variant |
| rs75821091 | IFI30 | 19 | G>A | 1.226 | 0.932-1.618 | 0.147 | none* |

* Its functional consequence has not been reported.
